# Prospects of complete feed system in ruminant feeding: A review

**DOI:** 10.14202/vetworld.2017.424-437

**Published:** 2017-04-21

**Authors:** Yasir Afzal Beigh, Abdul Majeed Ganai, Haidar Ali Ahmad

**Affiliations:** Division of Animal Nutrition, Faculty of Veterinary Sciences and Animal Husbandry, Sher-e-Kashmir University of Agricultural Sciences & Technology of Kashmir, Shuhama - 190 006, Srinagar, Jammu and Kashmir, India

**Keywords:** benefits, complete feed, concept, feeding, ruminants

## Abstract

Effective utilization of available feed resources is the key for economical livestock rearing. Complete feed system is one of the latest developments to exploit the potential of animal feed resources in the best possible way. The complete feed is a quantitative mixture of all dietary ingredients, blended thoroughly to prevent separation and selection, fed as a sole source of nutrients except water and is formulated in a desired proportion to meet the specific nutrient requirements. The concentrate and roughage levels may vary according to the nutrient requirement of ruminants for different production purposes. The complete feed with the use of fibrous crop residue is a noble way to increase the voluntary feed intake and thus animal’s production performance. In this system of feeding, the ruminant animals have continuous free choice availability of uniform feed mixture, resulting in more uniform load on the rumen and less fluctuation in release of ammonia which supports more efficient utilization of ruminal non-protein nitrogen. Feeding complete diet stabilizes ruminal fermentation, thereby improves nutrient utilization. This feeding system allows expanded use of agro-industrial by­products, crop residues and nonconventional feeds in ruminant ration for maximizing production and minimizing feeding cost, thus being increasingly appreciated. However, to extend the concept extensively to the field and make this technology successful and viable for farmers, more efforts are needed to be taken.

## Introduction

Livestock forms an integral part of agriculture sector and encompass a great impact on the national economy; however, nutritional inadequacy for livestock is at currently referred one of the most burning global problems of agricultural countries. Due to higher population growth rate and its consequential pressure, available land for forage production is declining day by day. This wicked scenario of quality feed resource availability has virtually eclipsed the genetic worth of animals. In such circumstances, the extensive use of crop residues in livestock feeding seems to be indispensable to meet the nutritional needs of livestock; however, the major constraint in the utilization of these crop residues is high cellulosic contents and poor nutritive value that even cannot support the maintenance nutrient requirement of the animals. Hence, efforts are being directed toward assisting the animals to utilize these low-grade feedstuffs more efficiently as effective utilization of available feed resources is the key to enhance livestock productivity economically. Efficient utilization of crop residues available locally in appreciable quantum seems to be accomplished by the application of feed technology to maximize advantage from feeds in animal system. In this direction, many technologies have been developed so far, but for several reasons, they have not been adopted by the end-users, especially those having limited finance and poorly skilled [1]. The complete feed system is one of the latest developments in this context to exploit the potential of locally available animal feed resources in the best possible way.

Balanced and economical feeding of livestock is extremely important for optimum productivity. Lower livestock production is mainly due to the scarcity of feeds and unbalanced feeding practices. To minimize feed costs and labor, and to maximize production is the need of the time and can be achieved by blending concentrate, mainly comprised locally available by-products and roughage portions of the ration to form complete feed/diet, synonymously termed as total mixed ration (TMR). Complete feed with the use of fibrous crop residue is a noble way to increase the intake and to improve feed utilization and animal production performance. The complete feed system is being increasingly appreciated as it allows expanded the use of agro-industrial by­products, crop residues and non-conventional feeds in livestock ration for maximizing production and minimizing feeding cost.

## Benefits of Complete Feed System

The merits of complete feed are related to stable rumen environment for optimum fermentation with minimal fermentation losses and fluctuation in the release of ammonia, better utilization of rumen non-protein nitrogen, and stabilization of the acetate to propionate ratio which favors normal fat synthesis and enhancement of utilization of low-grade roughages [2,3]. Feeding complete feed ensures better consumption, thereby reduces wastage. In this system, the high yielders are allowed to realize their potential enabling them to eat according to their yield while avoiding wastage by low yielder [4].

Complete feed avoids selective refusal of unpalatable dietary portions, thus has provision for incorporation of nonconventional feed resources to economize animal feeding. Waste materials - such as waste paper [5], cotton-seed hulls [6], cotton straw [7,8], lentil straw [9,10], sewen grass, mesquite pods, groundnut straw, tumba oilcake, taramira oil cake and groundnut hulls [11], forest grasses [12], sweet sorghum bagasse (SSB) [13-18], tree foliage [19-22], spent maize cobs [23], sugarcane bagasse pith [24], crop residues [25-27], poultry dropping [28], babul pods [29-31], olive cake [32], neem seed cake [33,34], karanja seed cake [34], and guar meal [35,36] - have been successfully incorporated in complete diets for livestock, thus lower feeding costs.

The complete feed has a balanced ratio of concentrate to roughage portions, reduces eating and rumination time, and thus increases resting time. The stall feeding system of livestock with complete feeds during climatic vagaries (drought, famine, and snow storms) has been advocated [1,37]. Sharma *et al*. [11] suggested that highly fibrous unconventional feedstuffs could be incorporated to form complete economic rations for sustaining sheep farming during feed scarcity in arid region. Singh *et al*. [27] indicated that goats could be maintained under stall feeding with complete diets containing high proportion of crop residues (gram or arhar straw). Furthermore, it has been reported that supplementation of the complete feed in block form during feed scarcity in semi-arid region helped grazing ewes to maintain their body weight (BW) compared to unsupplemented ewes [38]. Feeding complete feeds appear superior to grazing plus supplementation in terms of dry matter (DM)/nutrient intakes and animal performance, thus can be recommended as an alternative to such feeding strategy for sheep production, especially where the pastures are highly eroded and need resting for regeneration or curing [37,39].

## Feeding Complete Feed

Several studies have been carried out for assessing the effects of feeding complete feeds in different ruminant livestock species on various aspects as under:

### Effect of complete feed on *in vitro* fermentation characteristics

The chemical analysis of feedstuffs provides idea only about nutritive composition but does not give information about their fermentation values [40], which is important for ruminant digestion. *In vitro* rumen fermentation technique, a rapid method of analysis has widely been used to study feed degradation, detect small differences in fermentation characteristics between feedstuffs and allow more frequent sampling. This technique enables selection of feed or feed constituents for high efficiency of microbial protein synthesis in rumen along with high DM digestibility and provides a basis for the development of ruminant feeding strategies to maximize substrate fixation into microbial cells [41].

In *in vitro* studies, supplementation of complete feeds with different feed additives has been reported to improve the nutrient degradability by several workers. Mir *et al*. [42] observed increase in *in vitro* DM degradability (IVDMD) and *in vitro* organic matter degradability (IVOMD) on supplementation of different herbs at 2% and 3% level in complete feeds for goats. Ganai *et al*. [43] also reported that degradability of nutrients (DM, OM and neutral detergent fiber [NDF]), total gas production (TGP), total rumen nitrogen, and total volatile fatty acid (TVFA) concentration in *in-vitro* system was significantly improved when bajra straw-based complete feed was supplemented with Bhringraj herb at 3% DM in comparison to un-supplemented complete feed at 48 h incubation. In another experiment, Ganai *et al*. [44] again reported improvement (p<0.01) in IVDMD, IVOMD and IVNDFD as well as TGP on supplementation of yeast to bajra straw and bajra straw-based complete feed. Likewise, Nehra *et al*. [45] concluded that live yeast culture supplementation improved (p<0.01) IVDMD and IVOMD of dew bean straw-based complete feeds; besides, the straw could be optimally incorporated in the complete feed up to 60% level without any adverse effect. While determining the best possible level of thermotolerant yeast for sheep diet by *in vitro* fermentation experimentation, Harikrishna *et al*. [46] reported that level up to 3 g/kg could be incorporated in adult sheep rations economically. Similarly, incorporation of enzymatic extract of *Cellulomonas flavigena* in TMR increased *in vitro* degradation of cellulose from forages that are fed to ruminants [47]. Thakur and Shelke [48] assessed the effect of different periods of storage and temperatures of TMR containing exogenous fibrolytic enzymes (EFEs) on enzyme activity and *in vitro* digestibility and concluded that there was no adverse effect of storage (up to 60 days) and heating (up to 80°C) of TMRs on cellulose and xylanase activities and *in vitro* fiber digestibility. Grewal *et al*. [49] assessed the effect of different metabolizable energy (ME) (100%, 110% and 120% National Research Council) and undegradable protein (UDP) (24%, 32% and 40% of crude protein [CP] [16.8%]) levels on the *in vitro* fermentation of complete feeds and showed that the diet containing ME at 110 or 120% of NRC (2001) with low UDP (24% of dietary CP) supplemented with niacin gave the best response for nutrient digestibility and ME availability. Singh and Srinivas [50] assessed associative effect of cereals and oil cakes in concentrate supplements and complete diet for ruminants on *in vitro* fermentation kinetics and reported that *in vitro* gas production (IVGP) pattern of maize and oat-based concentrate supplement in complete ration mixture were comparable; however, the extent of IVGP on maize-based ration was better than sorghum-based ration and urea-mineral salt fortification yielded better improvement in fermentation pattern on maize-based ration than either sorghum or oat-based rations. Singh [51] showed that incorporation of slow release urea up to 30% of CP in TMR with niacin at 600 ppm level resulted in highest efficiency and maximum microbial mass production as compared to other replacement levels of CP (10% and 20%) and niacin in *in vitro* fermentation studies. Likewise, Grewal *et al*. [52] reported that combination of niacin and vitamin E supplementation improved the *in vitro* utilization of complete feed.

Beneficial effects of incorporating nonconventional feedstuffs in complete feeds on *in vitro* rumen fermentation pattern have been observed. Jakhmola *et al*. [20] reported that inclusion of *Calligonum polygonides* leaves in sewan (*Lasiurus sindicus*) grass-based complete diets for ruminants had a potential to positively affect rumen metabolism *in vitro*. Goswami *et al*. [35] reported that IVDMD and IVOMD decreased by replacing groundnut cake (GNC) with guar (*Cyamopsis tetragonoloba*) meal in concentrate portion of TMRs at all (25%, 50%, 75% and 100%) levels, but depression (p<0.05) was observed in TMR’s containing 50% or more replacement levels. Likewise, Ramachandra *et al*. [53] concluded that the pulse by-products (straw and chunnies) based complete diets with the different levels of rate of gas production (k) and partitioning factor could increase (p<0.01) the microbial biomass synthesis under *in vitro* fermentation.

### Effect of feeding complete feed on DM and nutrient intake

Increased intake of DM and nutrients in different ruminant species on complete feed system compared to conventional feeding system has been reported by several workers [7,8,10,12,25,54-59]. Complete feeding system is considered better over separate feeding system [60]. Complete ration has beneficial effects in terms of utilization of low-grade roughages; besides, its densification into feed blocks [19,26,61] or extrusion of the complete mash feed into pellets [7,8,12,14,17,27,62] distinctly improves the DM/nutrient intake and nutritive value of mash diet, thereby ensures efficient utilization of the feed [63]. A higher intake, therefore, gives scope for using cheap ingredient and various bulky by-products that are available locally to economize livestock feeding.

### Effect of feeding complete feed on nutrient utilization

Feeding complete diet stabilizes ruminal fermentation that improves efficiency of nutrient utilization in ruminants [3]. Feeding of ammoniated lentil (*Lens culinaris*) straw-based TMR to growing Barbari kids improved digestibility of various nutrients as well as DCP content (p<0.01) of the diet [9]. In another experiment, Mudgal *et al*. [10] reported improvement in digestibility of various nutrients as well as TDN content (p<0.01) of the diet for kids compared to those fed the straw *ad libitum*. Likewise, Kishore *et al*. [57] reported that crop residues (maize stover [MS], red gram straw and black gram straw) based complete rations provided better nutrient supply in terms of higher DCP and TDN values and intakes of DM/digestible nutrients/DE and ME to Nellore rams compared to conventional ration. Similar results have been reported by Sharma *et al*. [58] in calves fed complete feed in mash form compared to those fed the diet in conventional form.

Utilization of poor quality feedstuffs can be improved by their incorporation in complete diet rather than feeding separately along with concentrate mixture. However, adequate DCP content in crop residue-based complete feeds is necessary to meet the maintenance and growth requirements of the animals. Senani *et al*. [23] reported that ground maize cobs could replace ragi straw completely in the TMR for lambs without any significant effect on DM intake and nutrient digestibility. Kumar *et al*. [64] concluded that a dietary CP level of 13.5% in complete feed was sufficient for successfully and economically raising of calves with no significant differences for feed intake and utilization compared to other higher (15% and 16.5%) levels. The use of urea and molasses in paddy straw-based complete diets made from locally available ingredients might maximize and economize the production of livestock through improved degradability and utilization of nutrients and might be accessed through feeding trials in animals [65]. Likewise, Pandya *et al*. [66] reported similar intake of DM, CP, DCP and TDN as well as average daily gain (ADG) in crossbred calves fed complete feed based on wheat straw (30%) and conventional ingredients, complete feed based on sugarcane bagasse (30%) and nonconventional ingredients or conventional feed with higher protein utilization efficiency in both the complete diets fed groups. While evaluating three monsoon herbages processed into complete pellet feed for feeding to Barbari male kids during fodder scarcity periods, Tripathi *et al*. [67] reported that digestibility of DM, OM and CP was higher (p<0.05) in kids fed *Tephrosia purpurea* based diet possibly due to higher CP content and therefore intake, while cellulose digestibility was higher (p<0.05) in those fed *Dactylotennium aegypticum* based diet.

Processing of the complete feeds has been reported to improve the nutrient utilization in ruminant animals. Incorporation of crop residues as a roughage source in TMR for ruminants and their further processing (densification/pelleting) is one of the practical ways of improving their utilization. The physical form of the diet can affect potential rate of consumption with densified/pelletized diets ingested more rapidly than those in mash form. In an experiment carried out by Afzal *et al*. [19] on Corriedale lambs, significant (p<0.05) improvement in feed conversion efficiency with marked reduction in cost of production by feeding complete feed block has been reported, while DM/nutrient intake and digestibility of DM/gross nutrients, nutritive value and retention of major minerals did not change between the groups. However, Singh *et al*. [61] reported higher (p<0.05) feed intake in calves fed wheat and rice straw-based complete feed in block forms as compared to those fed mash forms of complete feeds, while the digestibility of DM and gross nutrients along with different nutrient intakes did not differ among the calves of dietary treatment groups. Furthermore, Ghosh and Chatterjee [68] reported that MS-based complete feed block could successfully replace the straw and tree leaves based diet supplemented with concentrate mixture in conventional form for yaks without any adverse effect on voluntary feed intake and nutrient digestibility during winter feeding. In contrast, Khan *et al*. [69] reported that physical form (mash vs. block) of the diet had no significant effect on nutrient digestibility in lambs except CP. Likewise, pelleting of complete diets has been reported to improve the voluntary feed intake and nutrient utilization to enhance animal performance. Waje *et al*. [12] reported higher intake and digestibility of nutrients in growing crossbred calves fed mixed forest grasses-based complete pellet feed as compared to calves fed either conventional or TMR diets. Another processing method for feeding complete diets is expansion-extrusion which also has proved to be better than feeding complete diet in mash or conventional forms. To identify the appropriate processing method for efficient utilization of SSB after blending with concentrate in complete diets for buffalo calves [14,17] and growing lambs [18], expander-extruded pellet complete diets were observed to be better in terms of nutrient utilization efficiency and microbial N supply than chopped or mash forms of the complete diets. The complete diet based on paddy straw incorporated at 50% level was more palatable to sheep when it was expander-extruder pelleted and such processing improved the nutritive value of the diet [70]. In contrast, Reddy *et al*. [62] reported that expansion-extrusion pelleting of complete diets significantly (p<0.001) increased the voluntary feed intake compared to the similar mash diets with no effect on digestibility of nutrients. Likewise, Datt *et al*. [59] reported that blending of paddy straw into complete diets significantly increased digestibility of protein, nitrogen balance and DCP and TDN intakes compared to conventional diet; however, expansion-extrusion processing of mash diet did not have additional advantage over mash diet.

Supplementation of feed additives in complete diets improves their nutritive value and nutrient digestibility in ruminant livestock besides have health promoting and feed economizing effects. Harikrishna *et al*. [46], while determining the optimum level of thermotolerant probiotic yeast in straw-based complete diet for lambs, reported that yeast had potential at 1-3 g/kg level in improving digestibility of nutrients, intake of DCP and ME, and N retention; however, incorporation of 1 g/kg level appears to be economical with fattening diets that may be beneficial for livestock producers. Futhermore, Bhima *et al*. [71] reported that supplementation of yeast at 0.1% level in straw-based complete diet improved the digestibility of DM, CF, Neutral Detergent Fiber NDF, acid detergent fiber, cellulose, hemicellulose as well as TDN and ME value of the diet without affecting DM/nutrient intakes and N balance in Murrah buffalo steers; however, beyond 0.1% of supplementation no significant effect on nutrient digestibility and intake were observed. Likewise, live yeast culture supplementation to green gram straw-based complete feed in block form improved nutrient utilization through increased ruminal bacterial count leading to increased rate of fiber digestion in the rumen as well as increased flow of microbial protein from the rumen [72]. In an experiment carried out by Sehgal *et al*. [73] to assess the effect of incorporation of fungal zoospores of *Orpinomyces joyonii* in paddy and wheat straw-based TMRs for lactating buffaloes, improvement (p<0.05) in digestibility of all the nutrients as well as the %TDN content of the diet has been reported.

The roughage:concentrate (R:C) ratio in complete feed may vary in accordance to the production performance of animals. Reddy *et al*. [62] reported that red gram straw could efficiently be utilized up to 50% level in expander-extruder pellet complete diets for growing goat kids. Ammoniated wheat straw to the extent of 70% in complete feed for buffalo heifers has been found to be more economical [74]. For growing lambs, the optimum level for incorporation of groundnut haulms/straw in complete feed was suggested to be 60% [75,76]. Likewise, Dhuria *et al*. [77] reported that mustard straw could be incorporated in complete feed for sheep up to 60% level without much adverse effect on voluntary feed intake and nutrient utilization. Moreover, it has been reported that SSB could be incorporated at 50% [15] while as sweet sorghum stover could be incorporated at 60% [78] level replacing conventional roughages such as sorghum stover and MS in complete diets for sheep. Similarly, Nagalakshmi *et al*. [79] reported that red gram stalks could be incorporated up to 40% level in complete diets for buffalo bulls, while incorporation of the same at 50% level depressed nutrient digestibility and lowered nutritive value of the diet. In an experiment carried out by Rajamma *et al*. [80] to study the effect of supplementation of EFEs in TMR for adult buffalo bulls on nutrient utilization, it has been reported that nutrient digestibility improved irrespective of the R:C ratio in the diet. Veerannapet *et al*. [81] reported that MS could be included at 50-60% level in mash complete ration for optimum nutrient utilization in male lambs. Sinha *et al*. [82] reported similar DM and OM intakes in buffaloes and cattle fed TMRs with different ratios (60:20:20, 40:30:30, and 20:40:40) of concentrate, wheat straw and green fodder; however, heat production and oxygen consumption were lower while urinary N excretion, respiratory quotient and methane emissions at similar level of DM intake were higher in buffaloes than that in cattle, with higher proportion of concentrates in the diet decreased methane emissions linearly in both the species.

Feeding complete diet provides scope for incorporation of nutritious non-conventional feeds resources in concentrate or roughage component by avoiding selective eating of palatable components by animals. Mishra *et al*. [21] reported that fallen tree leaves (FTL) as replacement to pearl millet stover could safely be used up to 20% level as a roughage component of complete feed in block form without any adverse affect on nutrient utilization efficiency and microbial N supply; however, increasing the level of FTL to 30% in the diet showed declining trend on nutritional performance parameters. In an attempt to replace concentrate mixture by leaf meal in complete feed for sheep, Ganai and Beigh [22] reported that concentrate mixture could be efficiently replaced by leaf meal up to 30% level with no adverse effect on nutrient utilization. Babul pods could be incorporated safely in TMRs of lactating [29] and pregnant [31] goats to the extent of 16.5% without affecting the nutrient utilization, N and macro mineral balances; however, could be utilized up to 25% in the diet after Ca(OH)_2_ treatment. Similarly, Samanta *et al*. [83] reported that *Leucaena* leaf meal could replace mustard cake in concentrate portion of complete diets up to 30% safely and inexpensively without affecting nutrient utilization in Barbari goats. In a study carried out by Bhatta *et al*. [84], while assessing the effect of feeding high tannin containing *Prosopis cineraria* leaves in complete feed mixture on the performance of lambs and kids, reported that feed efficiency ratio was the best in animals fed on complete feed containing 50% leaves. Tiwari *et al*. [24] reported that wheat straw could be completely replaced by urea ammoniated sugarcane bagasse pith in complete diet for crossbred bulls as source of dry roughage without any adverse effect on DM/nutrient intakes, digestibility of nutrients (CP and EE), and balances of N and major macrominerals (Ca and P). Olive cake, a highly fibrous feed is a better substitute to wheat straw. Ensiling process is a feasible means of converting and utilizing olive cake into reasonably nutritious feed when treated with urea. Such a product as part of complete ration could make a significant contribution to sheep production under intensive farming [36]. Likewise, Goswami *et al*. [35] reported no difference with respect to DM/nutrient intakes and digestibility of nutrients among crossbred calves fed TMRs prepared by replacing (50% and 75%) GNC with guar meal.

### Effect of feeding complete feed on growth

Feeding of small ruminants with crop residues-based complete rations appears to be the promising feeding system for improving their productivity in agricultural countries like India. Complete feed system improves nutrient utilization that supports higher growth performance and reduces the cost/kg live weight gain, thus is economical in comparison to conventional feeding system. Several studies have observed the beneficial effect of feeding complete ration on the growth performance of animals. Malisetty *et al*. [16] reported that Nellore lambs fed SSB-based complete diet had the ADG almost similar to those animals fed lucerne hay plus maize silage *ad libitum*. Nagi *et al*. [25] reported higher (p<0.01) ADG and nutrient utilization efficiency in Deccani lambs fed ground paddy straw-based complete diets compared to those fed conventional diet. Similarly, Chaturvedi *et al*. [37] reported that complete feed offered to Avikalin lambs under stall feeding system appeared superior to grazing plus supplementation system in terms of intake and animal performance. In another experiment, Chaturvedi *et al*. [38] observed that supplementation of complete feed in block form to grazing ewes during feed scarcity in semi-arid region helped in sustaining their BWs due to better nutrient availability compared to unsupplemented ewes maintained on sole grazing that lost 1.52 kg BW. Bartley [85] observed that feeding complete ration in pelleted form containing 25% sun-cured alfalfa hay to the calves significantly increased feed consumption and weight gain compared to calves fed milk starter and hay separately in conventional pattern. Florek and Lewicki [86] reported higher ADG and DCP intake in fattening bulls fed complete feed. Enhanced N balance and growth response in sheep fed complete rations containing legume straw has also been reported by Bonsi *et al*. [87]. Sorghum straw, soybean straw, and corn cobs-based complete rations could maintain adult Osmanabadi goats satisfactorily with DCP and TDN intakes higher than ICAR [88] recommendations with no loss in BW [89]. Similarly, Venkateswarlu *et al*. [90] reported the significant effect on growth rate (93.5-103.3 g/day) and feed conversion efficiency in lambs fed crop residue-based complete ration in comparison to traditional grazed animals (48.5 g/day) with no concentrate supplementation. Poor quality crop residues like groundnut haulms and red gram bhusa inclusion into complete feeds have also been reported to enhance intake without affecting digestibility, which resulted in improved growth performance and feed conversion efficiency in bucks [91]. However, Sharma *et al*. [58] reported no significant difference in ADG (509, 556 and 496 g/day) of crossbred calves fed either wheat straw *ad* *libitum* and concentrate mixture separately in conventional form or the wheat straw-based complete feed in mash and block form, although the calves fed complete mash feed gained higher BW than calves in other dietary treatment groups. The CP level of 15.4% in complete pellet feed has been reported beneficial for commercial goat farming under stall feeding system [92]. Likewise, Nagpal and Singh [93] observed comparable growth performance and feed utilization efficiency in 3.5-year-old camel calves on complete pellet diets containing either 8.34% or 10.40% CP and concluded that they could be successfully and economically raised on dietary protein level of 8.34% CP and 65% TDN.

The level of roughage source in complete feed can be variable depending on production performance of animals; besides, the animal response varies with R:C ratio. Ganai and Beigh [22] reported that replacing concentrate mixture by leaf meal in complete rations for sheep at 30% level resulted in best growth performance in terms of total gain and ADG of lambs. Prasad *et al*. [75] reported that the optimum level of groundnut haulms as sole roughage source in complete ration could be 60% without affecting the growth performances, feed efficiency and nutrient digestibility in growing lambs. Likewise, higher (p<0.05) nitrogen balances were observed in Nellore×Deccani lambs fed complete mash ration based on MS incorporated at 50% level compared to those fed MS-based rations included at 60% and 70% levels [81]. Mattoo and Ganai [94] reported highest weight gain in Corriedale sheep fed 40% roughage-based complete diet among the groups fed three iso-nitrogenous complete rations containing 40%, 50% and 60% roughage (rice straw and mixed tree leaves). Venkateswarlu *et al*. [95] concluded that inclusion of sorghum straw at 50-60% level in the complete ration could be practiced for feeding of growing lambs without affecting the growth rate. Furthermore, Dhore *et al*. [96] were of the same opinion that sorghum straw could be used as a sole roughage source at 60% level in complete pellet feed for intensive goat production. MS, a potential cereal crop residue hitherto unused could be incorporated as roughage component in complete rations at 50-60% level for obtaining optimum growth with better feed efficiency in growing lambs [97].

Inclusion of nutritious non-conventional but safe feed resources in concentrate or roughage component of the complete feed has proved to be beneficial for promoting the growth performance of ruminants. Ammoniation of lentil straw for use in TMR might be an effective way for improving nutrient utilization to get better growth response along with reduction in cost of feeding small ruminants [9,10]. SSB and leaf residues remaining after juice extraction could be used as a major component of complete feeds in diverse physical forms mitigating the threat of feed shortage [13]. Senani *et al*. [23] reported that ground spent maize cobs could replace ragi straw completely in the TMR of lambs without affecting the weight gain. Tiwari *et al*. [24] reported that ammoniated sugarcane bagasse pith could be used up to 50% in the complete diet of growing cattle bulls without any adverse on their growth performance; however, inclusion of the same at higher levels resulted in significant (p<0.05) reduction in growth rate. Kushwaha *et al*. [30,31] reported that babul (*Acacia nilotica*) pods might be incorporated up to 33% in concentrate mixture of TMR for pregnant and lactating goats without affecting growth rate and it might be increased up to 50% by treating with Ca(OH)_2_ before use. Neem (*Azadirachta indica*) seed cake could be incorporated into complete diet for lambs after detoxification without any deleterious effect on growth and meat quality [33]. Goswami *et al*. [35] reported improved live weight gain and feed conversion efficiency in crossbred calves fed TMR in which 50% GNC in concentrate portion was replaced with guar meal; while at 75% replacement, there was decrease in live weight gain, ADG and feed conversion efficiency; however, these negative effects were overcome by added sweetener in the ration. In another similar experiment, Sharma *et al*. [36] reported no adverse effect on nutrient utilization efficiency and thereby growth rate by feeding 5% guar meal as protein supplement in compressed complete diets to growing calves. Monsoon herbages (*D. aegypticum*, *Cenchrus ciliaris*, and *T. purpurea*) have potential for feeding to kids in the form of complete pellet feed during feed scarcity periods for supporting their optimum growth [67]. Conventional protein supplement alone or in combination with energy source with nutritionally improved poor quality crop residues like fermented wheat straw could be used as complete feed for growing animals in comparison to conventional feeding system, where roughage is supplemented with concentrate mixture [98]. Rekhate *et al*. [99] reported that arhar straw-based complete pellet ration could be fed to goats for optimum weight gain under intensive system of management. Similarly, tapioca leaves and tea waste (45:15) could be incorporated in complete pellet ration for growing kids with no significant effect on average weight gain and feed and protein conversion efficiencies compared to those fed guinea grass-based pellet ration as control [100]. Radhakrishnan and Balakrishnan [101] reported that neem (*A. indica*) leaves could be incorporated up to 40% level replacing groundnut haulms in complete rations without affecting the growth rate in kids.

Various studies have shown enhancement in growth performance of animals fed processed complete feeds. Feeding complete feed in block form to lambs resulted in higher ADG and N retention in comparison to those fed complete mash feed [8]. Furthermore, Singh *et al*. [61] reported higher (p<0.05) BW gain in calves fed rice straw-based complete feed blocks as compared to those fed mash form of the same complete feed. However, Ghosh and Chatterjee [67] reported no adverse effect on BW change in yaks during winter fed MS-based complete feed in block form compared to those fed straw and tree leaves based diet supplemented with concentrate mixture in conventional form. In a similar experiment, Medhi *et al*. [102] reported that feeding of complete feed in block form to lactating yaks during winter were comparable to silages with or without supplementation and had beneficial effects in terms of retention of loss in BWs compared to those on free grazing. The complete pellet feed is a technology to ensure proper nutrient supply and optimum production from the domestic animals. Waje *et al*. [12] reported that pelleting of complete ration improved (p<0.05) the growth rate of crossbred calves as compared to those fed either conventional or TMR diets; however, the cost of feeding/kg BW gain remained comparable. Likewise, Singh *et al*. [27] reported that feeding crop residues-based complete pellet diet improved feed conversion efficiency and reduced cost of feeding over complete mash diet in goat kids. Madhavi *et al*. [33] reported higher (p<0.05) ADG and lower cost per kg gain in Nellore lambs fed complete pellet diets in comparison to those on mash diet with no significant differences for carcass characteristics. Nagpal [103] reported that camel calves could be fed on complete pellet diet with 9.94% CP and 63.35% TDN for better growth rate and feed conversion efficiency. In addition, it has been reported that incorporation of urea at 1% in roughage-based complete pellet diets for growing camels could positively improve nutrients intake, digestibility, growth performance, and feed conversion efficiency [104]. Expander-extruder processing of roughage-based complete diets improves nutrient utilization efficiency, results in better growth performance and may form an economic ration for growing ruminants. Nagalakshmi and Reddy [8] assessed on farm performance of lambs and reported higher (p<0.01) ADG and feed utilization efficiency, and lower (p<0.01) cost of feed per kg gain in animals fed cotton stalks based expander-extruder processed complete diet in comparison to those fed conventional ration. SSB [14] or sweet sorghum crushed residue [17] might be economically used as an alternative roughage source to sorghum straw and feeding of complete rations in the form of expander-extruder pellets proved superior over chopped and mash forms of the same ration in terms of higher (p<0.01) ADG, lower (p<0.01) FCR and cost per kg gain. Furthermore, Reddy *et al*. [62] reported that expander-extruder pelleting of complete diets significantly increased the weight gain in goats.

Feed additives supplementation in complete diets improves nutrient utilization promotes higher growth rate in animals. The addition of an enzymatic extract of *C. flavigena* to forage in TMR did not improve digestion, and thus productive performance in lambs at the doses (0, 5.0 and 7.5 mL of extract per kg DM of TMR) evaluated [47]. However, incorporation of superior anaerobic fungal (*Neocallimastix* sp. GR-1) zoospores in wheat straw-based complete feed for Murrah male buffalo calves significantly increased nutrient digestibility and DE value that enhances feed efficiency (by 28.7%) and BW gains (by 16%) [105].

### Effect of feeding complete feed on quantity and quality of milk

Blending of roughage and concentrate portions into complete diet increases its energy density [3] and provide all the nutrients in required proportions, thus improve performance of dairy animals [106-109]. Besides, the physiological and economic problems encountered with *ad libitum* feeding of both the roughage and concentrate portions of the ration separately get reduced. Maximum benefits can be realized by incorporating such feeds as ingredients that are most economically purchased, transported and stored. Nagalakshmi and Reddy [8] in one on-farm trial reported higher (p<0.01) milk yield and feed cost per kg fat corrected milk (FCM) in buffaloes fed cotton stalks based expander-extruder processed complete diet compared to those fed conventional diet. In addition, Medhi *et al*. [102] reported that during winter, lactational performance of yaks fed complete feed in block form was higher (p<0.05) compared to those on free grazing. Feeding complete feeds significantly increased (p<0.05) milk production in buffaloes [107] and crossbred cows [109] as compared to conventional rations. Holter *et al*. [110] reported that feeding with the blended diet resulted in more milk with higher efficiency of ME utilization for milk production. Das *et al*. [111] reported higher average milk yield in complete feed block fed than in mash fed lactating buffaloes during an on-farm trial and farmers were of opinion that complete feed blocks not only enhanced milk yield but were also easy at feeding and storage. However, Rakes [112] concluded that complete rations containing 13-14% CP supported milk production at par with that obtained with conventional ration in dairy animals. Furthermore, Kumar *et al*. [113] reported that feeding of TMR to lactating buffaloes had no significant effect on milk yield compared to those fed the diet in conventional form.

Feeding of complete diets to dairy animals has variable results on milk composition. Nagalakshmi and Reddy [8] reported higher (p<0.01) milk fat and 6% FCM yield in buffaloes fed expander-extruder processed complete diet compared to those fed conventional diet in one on-farm trial, with no effect on milk solid-not-fat (SNF) content. In attempts to include non-conventional feed resources in the ration of dairy animals, it has been reported that babul pods could be included in the TMR of lactating goats safely up to 16.5% and even at higher levels up to 25% in diet after Ca(OH)_2_ treatment without affecting milk production and its composition [30]. Similarly, Raj *et al*. [34] concluded that detoxified neem and karanja cake could be incorporated in TMRs of medium producing dairy cattle (5-8 L milk/day) replacing standard soybean meal without adversely affecting milk composition and milk production efficiency in crossbred dairy cows. Kumar *et al*. [113] reported higher SNF, protein, total solids and polyunsaturated fatty acid content in milk of buffaloes fed TMRs compared to those fed the diet in conventional form. Likewise, Villavicencio *et al*. [114] found no significant difference in milk production or milk composition (except SNF) of high-producing cows fed complete feeds during long-term studies. O’Neil *et al*. [115] reported increased level of protein content in milk on TMR feeding as compared to separate feeding of roughages and concentrate.

R: C ratio in the complete diet for dairy animals is an important factor regulating the quality of milk. The most common dietary cause of low fat in milk is the diet containing a low level of forage and/or a high level of concentrate. The low milk fat is often confounded by the fact that low forage diets are often associated with high milk production which itself tends to lower fat %. Only 20% roughage in a complete diet could cause a cow to secrete milk with low-fat content, while 40% roughage level in complete ration produced significantly more FCM and milk fat than that of a 30% roughage ration with no significant differences in SNF [116,117]. Improvement in the total and FCM yield was noticed in buffaloes given a complete pellet diet containing 30% roughage [118]. Nelson *et al*. [119] reported that FCM production and % SNF significantly decreased among animals consuming the all forage ration compared to other rations, and % milk fat also showed highly significant linear decrease with increase in concentrate portion in complete ration. Owen *et al*. [120] fed dairy cows on complete diets containing various levels of milled straw and concluded that complete diets should contain a minimum of 24% milled straw. The optimum R:C ratio was not likely to exceed 60:40, with 50:50 ratio adequate for high yielding cows. With the increased level of straw from 20% to 50% in the complete diet for dairy cows, the fat percentage increased progressively while milk yield and SNF declined [121].

### Effect of feeding complete feed on rumen fermentation and hemato-biochemistry

Feeding complete feed to ruminants results in more uniform load on the rumen which is associated with less fluctuation in the release of ammonia so that rumen non-protein nitrogen is more efficiently utilized [3]. Complete rations comprising of locally available crop residues as roughage source on feeding provide conducive rumen environment for better nitrogen utilization and higher TVFA production than conventional ration [2]. Besides, the activities of different ruminal enzymes responsible for fiber degradation have been reported to be higher in animals fed complete ration as compared to animals fed conventional ration [122].

Several workers have observed no adverse effects of feeding different unconventional feedstuffs based complete rations on rumen fermentation. Feeding ammoniated lentil (*L. culinaris*) straw-based TMR to growing Barbari kids resulted in higher (p<0.05) total and ammonia-N with comparable pH and TVFA contents in rumen fluid compared to those fed untreated straw-based TMR [9]. However, Mudgal *et al*. [10] reported reduction (p<0.05) in pH and improvement (p<0.05) in TVFA production with comparable total and ammonia-N in kids fed ammoniated lentil straw-based TMR compared to those fed the straw *ad libitum*. Sharma *et al*. [11] reported no significant effect among the feed sources on rumen fermentation pattern in sheep fed various complete rations based on unconventional feed resources of arid zone of India. Mishra *et al*. [21] reported that inclusion of FTL to replace pearl millet stover in complete feed block diets for sheep increased ammonia-N, total-N and ciliate protozoa population with no effect on VFA concentrations. Likewise, replacing ragi straw with ground spent maize cobs at 50% and 100% level in the complete diets for lambs resulted in non-significant effect on various rumen fermentation metabolites; however, led to more favorable values for rumen metabolites along with increase in bacterial and fungal counts in the rumen [23]. Narasimha *et al*. [28] reported that the H^+^ ion and TVFA concentrations were significantly (p>0.01) higher in sheep than in goats while the concentration of total N, trichloracetic acid (TCA) insoluble N, food and protozoal N in SRL was significantly higher in goats than in sheep; however, ammonia and residual N concentrations did not differ significantly between sheep and goats by feeding complete mash diet containing poultry litter (35%). Herbages (*C. ciliaris, T. purpurea, D. aegypticum*) could be conserved during monsoon season for feeding to the growing kids as complete pellets diets during fodder scarcity periods without any adverse effect on rumen fermentation characteristics [67]. Likewise, Ramachandra *et al*. [53] reported that pulse by-products based complete diets could significantly (p<0.01) increase the rumen microbial biomass synthesis and suggested that the same could be tested for their microbial biomass efficiency *in vivo*.

Feeding complete feeds in different processed forms has been reported to be beneficial in animal system with respect to rumen fermentation by several workers. Complete pellet diet feeding causes changes in rumen fermentation pattern predominantly increases propionate production favoring glucose production for energy yield which is efficiently utilized for cellular growth and development, thereby reduces feeding/production cost of growing animals [123]. Significant (p<0.01) variations among all the studied rumen metabolites were observed in goats fed arhar straw-based complete pellet ration in comparison to those offered sole arhar straw pellets with concentrate pellet supplementation [100]. Datt *et al*. [59] reported significantly (p<0.01) higher concentration of total N, TCA precipitable-N, ammonia-N and TVFA in rumen liquor of sheep fed expander-extruder processed paddy straw-based complete diet than those fed the diet in conventional form. Likewise, Nagalakshmi and Reddy [70] reported lower (p>0.05) ammonia-N and higher total-N in sheep fed processed paddy straw-based complete diets than those fed the diet in mash form, while, TVFA concentration was higher (p<0.01) in SRL of lambs fed expander-extruder pellet diet. No adverse effects on rumen fermentation pattern and hemato-biochemical parameters were observed in sheep fed bajra straw [124], gram straw [125], and mustard straw [126] at 60% level based complete feed in mash and block form.

Supplementation of feed additives in complete feeds promotes rumen fermentation in a favorable direction to enhance animal performance. Nehra *et al*. [72] concluded that live yeast (*Saccharomyces cerevisiae*) culture supplementation to green gram straw-based complete feed blocks promoted rumen fermentation which improved nutrient utilization resulted in stimulated growth of kids. Likewise, yeast supplementation to groundnut straw-based complete feed block could be beneficial to improve the performance of sheep due to a marked improvement in rumen fermentation pattern [127]. Sehgal *et al*. [73] reported improvement (p<0.05) in various rumen fermentation parameters (TVFA, total-N, ammonia-N, TCA perceptible-N and total no of zoospores/mL SRL) in lactating Murrah buffaloes fed fungal (*O. joyonii*) zoospores incorporated paddy and wheat straw-based TMRs. Likewise, Kumar *et al*. [105] reported enhancement of nutritive value of wheat straw-based complete feed blocks with the incorporation of elite fungal zoospores through improvement in studied rumen metabolites (total-N, TCA-N, TVFAs, number of fungal zoospore and bacteria/mL SRL). Furthermore, Jha *et al*. [128] reported similar findings in buffalo calves fed wheat straw-based complete feed blocks incorporated with fungal (*Neocallimastix* spp.) zoospores and sulfur (1% sodium sulfate). This technology developed to improve the nutritive value of wheat straw-based diets, can be used at the compound feed industry level for providing a well-balanced wheat straw-based complete feed rations to the animals at farmers door.

Complete ration is the best feeding model as it reduces problems of nutrient deficiencies in livestock fed on poor quality feed resources with no adverse effects on animal health by allowing a synchronous and fractionated supply of all essential nutrients for attaining maximum production potential. Sharma *et al*. [58] reported no significant difference in serum glucose, total protein, albumin and globulin concentrations among the crossbred calves fed the diet in conventional form or the complete feed in mash and block form, whereas serum total cholesterol and SGPT (p<0.05) and alkaline phosphatase (p<0.01) activities were significantly less and SGOT activities were considerably less in calves fed complete feeds in mash or block form compared to those fed the diet in conventional form. In addition, Singh *et al*. [61] reported comparable blood biochemical values in crossbred calves fed wheat and rice straw-based complete feed either in mash or block forms. Comparable hemato-biochemical parameters were recorded in growing Sahiwal calves fed on TMRs with three (13.5%, 15% and 16.5%) levels of proteins and concluded that a dietary level of 13.5% CP for calves is sufficient to maintain blood parameters within the normal range [64]. Tripathi *et al*. [67] concluded that monsoon herbages might serve as good roughage source for kid production if fortified appropriately with concentrate feeds to form complete pellet feed resulting no change in their hemogram. Blood biochemicals were within the normal physiological ranges in all the goats with no alterations in protein utilization and thus blood glucose, plasma urea-N and total serum proteins fed complete diets based on natural grass hay and concentrate mixture with replaced mustard cake by *Leucenea* leaf meal [83]. Nagpal and Singh [93] observed comparable levels for blood glucose, total protein, albumin, triglycerides, cholesterol and creatinine in camel calves fed complete pellet diets containing either 8.34 or 10.40% CP, while concentrations of serum urea, Ca and P were higher (p<0.05) in animals of group fed at 10.40% CP level. Similar results have been reported for male camel calves fed roughage-based complete pellet diets with different CP and TDN values [103]. Kumar *et al*. [113] also reported that the levels of blood glucose, blood urea nitrogen (BUN), β-hydroxybtyrate, non-esterified fatty acids and total immunoglobulin in lactating Murrah buffaloes were not influenced either by separate feeding of roughage and concentrate or TMR feeding. No difference has been reported in BUN level between the goats fed complete ration or concentrate and forage separately [129]. However, Gupta *et al*. [122] reported that feeding of complete ration to crossbred cattle in contrast to conventional feeding caused changes in blood biochemical parameters indicating improvement in nutrient utilization. Likewise, Delany *et al*. [130] reported higher blood glucose concentration in cows fed TMR as compared to those grazed in pasture and supplemented with concentrate. In addition, Nagpal *et al*. [131] observed improvement in serum bio-chemicals (blood glucose and serum proteins) by feeding complete ration to camel calves resulting in their better health status. Rekhate *et al*. [132] reported significant effect of feeding complete pellet diet on various blood biochemicals in goats, although all were in the normal ranges. Beigh and Ganai [133] concluded that leaf meal mixture could be used as alternative protein source to replace part (30%) of conventional concentrate in TMR for raising lambs economically without any adverse effect on blood biochemicals and rumen metabolites. Ramulu *et al*. [134] concluded that dietary Zn supplementation of 140 ppm to buffalo calves in sorghum stover based complete diets could be recommended for obtaining better immune response with no adverse effect on hematological constituents and other essential minerals availability.

In spite of such great importance of complete feed system in ruminant feeding, the technology has remained confined to the organized farms only, as it is not economically feasible for small and marginal livestock farmers to purchase and operate the costly machinery required for blending the ingredients. As such, there is a dire need to extend the concept extensively to the field. In this direction, some limited efforts have been made by providing small-scale forage disintegrator and grinder cum mixer to the villages through local self-help groups on a community basis. Thus, to make this technology successful and viable for farmers, more efforts are needed.

## Conclusion

Complete feed system for feeding ruminants is comparatively better option than conventional feeding of concentrates and roughages separately or grazing plus supplementation. This has advantages in provision of balanced diet to the ruminants and helping better utilization of the locally available feed resources, resulting in higher productivity along with reduction in feed cost and labor. Thus, the concept of complete feed system is becoming increasingly popular. However, lots of efforts are still needed to be taken for extending the concept extensively to the field.

## Authors’ Contributions

This review is a part of PhD thesis of the first author YAB, who carried out the research under the supervision of AMG. HAA helped in thorough revision of the manuscript. All authors have read and approved the final version of the manuscript.
